# Coarctation of the Aorta and Late-Onset Sepsis With Supraventricular Tachycardia: A Clinical Case Review

**DOI:** 10.7759/cureus.65634

**Published:** 2024-07-29

**Authors:** Sakshi Upendra Bhatia, Pallavi Yelkur, Hamritha Ashok, Akshai R, Vaanmathi Azhagar Nambi Santhi

**Affiliations:** 1 Paediatrics, Saveetha Medical College and Hospital, Saveetha Institute of Medical and Technical Sciences (SIMATS), Saveetha University, Chennai, IND

**Keywords:** electrocardiogram, supraventricular tachycardia, sepsis, infant, coarctation

## Abstract

We report a case of an 11-day-old male infant who presented to our hospital with complaints of breathing difficulty and cough for two days prior to admission. The child had a seizure post-admission which was controlled with phenobarbitone. The child was connected to a mechanical ventilator. The baby also had persistent tachycardia, which upon doing an electrocardiogram (ECG) led to a diagnosis of supraventricular tachycardia (SVT). Additional investigations revealed the existence of late-onset sepsis (LOS), which was treated with appropriate antibiotics. In view of weak femoral pulses with normal brachial pulses, a diagnosis of coarctation of the aorta (CoA) was entertained. The diagnosis was confirmed by an echocardiogram, and the baby was transferred to a higher-level medical center for surgical correction. Regrettably, the baby succumbed to heart failure and shock on the 12th day of life. This case highlights the infrequency of CoA accompanied by SVT. The case delves into the challenges of diagnosing the condition, the necessary medical interventions, and the unforeseen complications that must be considered to reduce mortality in such circumstances.

## Introduction

A distinct aorta constriction that obstructs blood flow is known as coarctation of the aorta (CoA) [1]. Italian anatomist Giovanni Morgagni originally described CoA in the eighteenth century. In 1944, Dr. Crafoord carried out the first therapeutic surgical operation for this illness [2]. Congenital heart disease (CHD) causes approximately 28% of all significant congenital abnormalities [3]. Global estimates place the prevalence of CHD at eight per 1,000 live births [4]. CoA, which has an approximate frequency of four per 10,000 live births, accounts for 6%-8% of all CHDs [1]. The rate of prenatal diagnosis of CoA has increased due to advancements in fetal echocardiography, but the diagnosis is still difficult to make in utero [1]. Most cases of CoA are not frequently recognized at birth. Some infants may display signs such as breathing difficulty, pale complexion, recurring lower respiratory infections, and failure to thrive. Most patients may not receive a diagnosis until they go into congestive heart failure (CHF) or hypertension, a condition more common in older children. CoA carries a guarded prognosis if not promptly diagnosed and treated. Failure to do so can result in complications such as early onset of coronary artery disease, impaired ventricular function, formation of aortic aneurysms or dissections, and cerebral vascular disease. If severe coarctation occurs in infants, it can result in shock and potentially fatal consequences if not rapidly identified and treated [2,3]. Furthermore, if an infection, such as late-onset sepsis (LOS), coexists with CoA, the prognosis worsens. Risk factors for LOS include prematurity, low birth weight, mechanical ventilation, administration of parenteral fluids, poor hand hygiene, and invasive treatments by healthcare workers, such as central venous catheters [2-5]. One of the most frequent disorders in neonates requiring emergency cardiac care is supraventricular tachycardia (SVT). Any tachycardia that requires participation from at least one supraventricular structure above the His bundle's bifurcation in order to continue is known as SVT [6]. According to research, one in every 250-1000 pediatric patients will develop SVT [7]. Hinkle et al. conducted a retrospective multicenter cohort research on 103 individuals diagnosed with fetal SVT. Of this group, 37% had refractory SVT, and they were also more likely to go in for premature delivery [8]. Here, we report an extremely unusual case of an 11-day-old infant with CoA who presented with SVT.

## Case presentation

**Figure 1:**
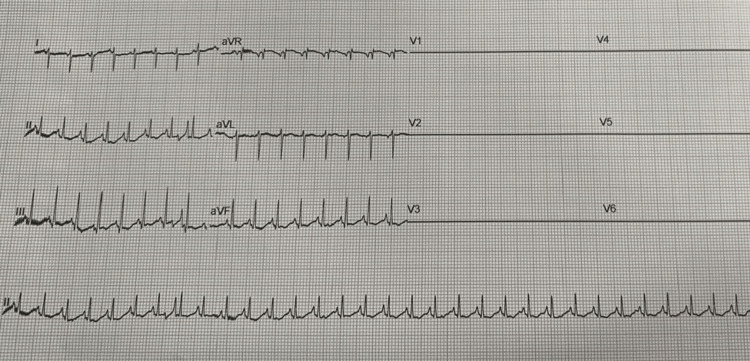
Electrocardiogram before administering injection adenosine 6 mg intravenously stat followed by injection amiodarone

**Figure 2:**
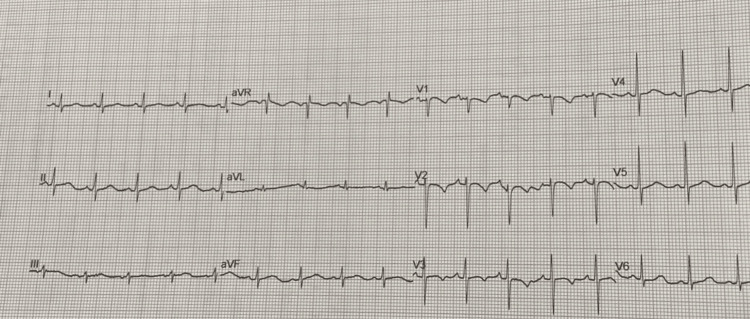
Electrocardiogram after administering injection adenosine 6 mg intravenously stat followed by injection amiodarone

An 11-day-old male infant presented to our hospital with breathing difficulty and cough for two days with no history of fever or feeding difficulty. On examination, the baby was found to be lethargic with a heart rate of 200 beats per minute and a respiratory rate of 80 breaths per minute. The capillary refill time was found to be prolonged. The child was maintaining a room air saturation of 85%. During examination, the child had an active seizure. The airway was secured, and the child was connected to a mechanical ventilator. A loading dose of intravenous phenobarbitone was given along with intravenous calcium gluconate. The baby continued to have persistent tachycardia. The electrocardiogram (ECG) revealed an absence of "P" waves and a narrow QRS complex tachycardia suggestive of SVT as illustrated in Figure 1. An immediate injection of adenosine (6 mg intravenously stat) was administered, followed by an injection of amiodarone, which led to the normalization of the ECG as illustrated in Figure 2.

Blood investigations revealed elevated total counts with a positive C-reactive protein indicative of sepsis as in Table 1. The venous blood gas revealed a picture suggestive of metabolic acidosis as in Table 2. The baby was started on intravenous antibiotics and oral propranolol. Upon further examination, feeble bilateral femoral pulses with normal brachial pulses were appreciated which led to the suspicion of CoA The ECG showed a severe post subclavian CoA with a coarctation segment measuring 2 mm and a moderate peri-membranous ventricular septal defect with a left-to-right shunt. Despite extraordinary measures, the child succumbed to life the next day due to cardiac failure.

**Table 1:** Laboratory investigations at the time of admission

Parameter	Observed value	Reference range
Hemoglobin	13.7 g/dL	15-24 g/dL
White blood cell count	13900 cells/µL	9100-34000 cells/µL
Platelet count	2.96 lakhs/mm³	0.84-4.78 lakhs/mm³
C-reactive protein	20.6 mg/l	<10 mg/l

**Table 2:** Venous blood gas at the time of admission pO_2_: Partial pressure of oxygen; pCO_2_: partial pressure of carbon dioxide

Venous blood gas	Observed value	Reference range
pH	7.2	7.35-7.45
pO_2_	30.7 mm hg	83-110 mm hg
pCO_2_	42 mm hg	83-110 mm hg
Bicarbonate	15.9 mmol/l	22-29 mmol/l

## Discussion

The presentation of CoA varies based on the age of presentation. It may include symptoms such as difficulty in breathing, difficulty in feeding, forehead sweating, and a pale appearance of the skin. Clinical examination findings include strong pulses and hypertension in the upper limbs contrasted by weak femoral pulses and low blood pressure in the lower limbs. Regardless of the severity of CoA, patent ductus arteriosus (PDA) aids with the perfusion of the lower body; therefore, newborns and neonates are typically asymptomatic immediately after birth. Upon birth, neonates with severe or critical cardiomyopathy experience symptoms of cardiogenic shock due to the closure of the ductus arteriosus [9]. If cardiac failure doesn’t occur in infancy, it is unlikely to occur at an older age. Prenatal diagnosis facilitates prompt therapy, prevents postnatal cardiac crises, aids delivery planning, and provides parental counseling [10]. Studies commonly acknowledge surgery as the first intervention of choice for substantial coarctation in neonates and early babies. The vast majority of surgeries performed today involve both end-to-end and extended end-to-end anastomosis procedures [11,12]. Following repair, surgical mortality is comparatively low. High blood pressure, repeated damage to the laryngeal nerve, bleeding, subclavian steal syndrome, and residual coarctation can all make the first few days after surgery more difficult [13]. In newborns, the primary cause of the higher risk of sepsis is their underdeveloped immune system. Neonatal neutrophils, macrophages, or T lymphocytes do not provide a full inflammatory response owing to their immature function. Moreover, newborns are born with restricted amounts of immunoglobulins and lack the capacity to produce an adequate mounting response against pathogenic organisms, either quantitatively or qualitatively [14]. LOS usually presents after 72 hours of life with septicemia, pneumonia, or meningitis. They may present with nonspecific symptoms like respiratory distress, hypothermia or hyperthermia, lethargy, or shock. In developing countries, it can be avoided by maintaining good hygiene, avoiding bottle feeds, and pre-lacteal feeds. Sometimes, laboratory results showing hyperglycemia, hypoglycemia, acidosis, or hyperbilirubinemia support the diagnosis. The presence of cardiac failure in this case further aggravated the shock. 

In a study conducted by Garson et al., 217 children with SVT were reviewed. Congestive cardiac failure accompanied SVT in 38% of the patients in this study. The majority of newborns are less than a month old, while the peak incidence of SVT presentation in children is under one year old. A total of 23% of the patients included in this study had a diagnosed CHD [15]. Yang et al. reported a case of an 18 years old who was a known case of hypertrophic cardiomyopathy (HOCM) and primary hypertension who presented with SVT. Failure of the ablation procedure via the femoral artery led to the possibility of CoA complicating the case which was confirmed by ECG [16]. Following a trial of vagal maneuvers, the American Heart Association's Pediatric Advanced Life Support Guidelines recommend adenosine [17]. An infant with CoA and SVT, complicated by LOS, presented to be a rare case. Although surgical correction was planned, the baby developed cardiac failure with shock and eventually succumbed to death.

## Conclusions

SVT often presents in neonates as congestive cardiac failure. Children with CHDs are at a higher risk of such arrhythmias than the general population. Early detection and immediate action are crucial for providing optimal treatment to these children. However, unanticipated complications such as unfavorable late-onset newborn sepsis have led to the infant's development of heart failure and shock, ultimately resulting in his death on the 12th day of life. This case emphasizes the importance of a good clinical examination at birth including looking for the equality of upper and lower limb pulses and a variation in blood pressure between the upper and lower body which may lead to an earlier diagnosis of conditions like CoA where a routine echocardiogram is not done for all children. It also stresses the rarity of CoA with SVT and discusses the diagnostic challenge, medical treatment, and unforeseen complications that should be considered in order to limit mortality in such cases. 
